# Allatotropin: An Ancestral Myotropic Neuropeptide Involved in Feeding 

**DOI:** 10.1371/journal.pone.0077520

**Published:** 2013-10-15

**Authors:** María Eugenia Alzugaray, Mariana Laura Adami, Luis Anibal Diambra, Salvador Hernandez-Martinez, Cristina Damborenea, Fernando Gabriel Noriega, Jorge Rafael Ronderos

**Affiliations:** 1 Cátedra Histología y Embriología Animal, Facultad de Ciencias Naturales y Museo; Universidad Nacional de la Plata (FCNyM -UNLP), La Plata, Argentina; 2 División Zoología Invertebrados, Facultad de Ciencias Naturales y Museo; Universidad Nacional de la Plata (FCNyM-UNLP), La Plata, Argentina; 3 Centro Regional de Estudios Genómicos, Universidad Nacional de la Plata (CREG-UNLP), La Plata, Argentina; 4 Centro de Investigación sobre Enfermedades Infecciosas, Instituto Nacional de Salud Pública (CISEI-INSP), Cuernavaca, Mexico; 5 Department of Biological Sciences, Florida International University, Miami, Florida, United States of America; University of Rouen, France

## Abstract

**Background:**

Cell-cell interactions are a basic principle for the organization of tissues and organs allowing them to perform integrated functions and to organize themselves spatially and temporally. Peptidic molecules secreted by neurons and epithelial cells play fundamental roles in cell-cell interactions, acting as local neuromodulators, neurohormones, as well as endocrine and paracrine messengers. Allatotropin (AT) is a neuropeptide originally described as a regulator of Juvenile Hormone synthesis, which plays multiple neural, endocrine and myoactive roles in insects and other organisms.

**Methods:**

A combination of immunohistochemistry using AT-antibodies and AT-Qdot nanocrystal conjugates was used to identify immunoreactive nerve cells containing the peptide and epithelial-muscular cells targeted by AT in *Hydra* *plagiodesmica*. Physiological assays using AT and AT- antibodies revealed that while AT stimulated the extrusion of the hypostome in a dose-response fashion in starved hydroids, the activity of hypostome in hydroids challenged with food was blocked by treatments with different doses of AT-antibodies.

**Conclusions:**

AT antibodies immunolabeled nerve cells in the stalk, pedal disc, tentacles and hypostome. AT-Qdot conjugates recognized epithelial-muscular cell in the same tissues, suggesting the existence of anatomical and functional relationships between these two cell populations. Physiological assays indicated that the AT-like peptide is facilitating food ingestion.

**Significance:**

Immunochemical, physiological and bioinformatics evidence advocates that AT is an ancestral neuropeptide involved in myoregulatory activities associated with meal ingestion and digestion.

## Introduction

Cell-cell interactions are a basic principle for the organization of tissues and organs allowing them to perform integrated functions and to organize themselves spatially and temporally. Molecules involved in cell-cell recognition, as well as intercellular messengers, appeared early on in evolution. In fact, cellular messengers are not only present in multicellular organisms, but are also present in unicellular eukaryotes such as the insulin-like peptides of *Tetrahymena pyriformis*, *Neurospora crassa* and *Aspergillus fumigate* [1]. The expression of immunoreactivity against RFamide neuropeptide antibodies has been shown in the phylum Placozoa, which is probably the most ancestral group among metazoa - having no organs or specialized tissues- [2]. Non-peptidergic messengers such as epinephrine and norepinephrin have been found in Porifera [3,4] and Cnidaria [5]. In addition, serotonin has been reported in Hydrozoa, where it seems to be involved in the onset of metamorphosis [6]. 

Cnidaria is the most ancient group of animals with differentiated tissues, represented by close to 10,000 existing aquatic species. They are located near the root of metazoan evolution and are likely the first group of animals exhibiting a nervous system [7]. In fact, two types of nerve cells have been described, corresponding to sensory and motor neurons [8]. The presence of several peptidic cell messengers have been described, suggesting that peptides were the first type of molecules acting as neurotransmitters or neurohormones in the course of evolution [7]. 

Peptides are pleiotropic molecules with a diverse range of functions. In Hydrozoa neuropeptides induce metamorphosis [6,9] as well as differentiation of neural stem cells to neurons [10]. Neuropeptides in cnidarians are also associated with regenerative processes [11-13] (for review see also 14) and myoregulatory functions with both excitatory and inhibitory activities [7,14-18]. 

Nervous and endocrine systems emerged relatively early on in the history of animal evolution. Molecules, either acting as messengers with similar functions or having evolved new functions, are present in both ancestral and derived groups. For instance, peptides sharing sequence similarity with vasopressin and endothelin, two peptides originally described in vertebrates as potent vasoconstrictors, have been also found in Hydrozoa associated with nerve cell differentiation [19], developmental processes and muscle contraction [20].

Allatotropin (AT) is a peptide originally isolated from the nervous system of the lepidopteran *Manduca sexta* because of its ability to stimulate the secretion of juvenile hormone by the *corpora allata* [21]. The presence of AT was later described in numerous hemimetabolous and holometabolous insect species [22-28]. AT has pleiotropic functions; it inhibits ion transport in the midgut of *M. sexta* larvae [29], as well as it controls the release of digestive enzymes in the midgut of *Spodoptera frugiperda* [30]. In addition, the myoregulatory and cardioacceleratory role of AT has been demonstrated in various insect species [31-39], where it is secreted not only by the nervous system, but also by endocrine epithelial cells, acting in a paracrine and endocrine manner [36-38,40,41]. Finally, it has been also proposed that AT is involved in the control of circadian rhythms [40,42,43].

Allatotropin related peptides have been reported in other phyla beyond Arthropoda. In fact, genes codifying peptides sharing similarity with AT have been reported in the limpet *Lottia gigantea* (Gastropoda: Mollusca) and in the worms *Capitella teleta* (Polychaeta: Annelida) and *Helobdella robusta* (Hirudinea: Annelida) [44,45]. Recently, we have demonstrated the expression of an AT-like peptide in three species of free living flatworms (Platyhelminthes) from different groups of turbellaria (Catenulida, Macrostomorpha and Rhabdocoela), where the peptide is present in neurons morphologically and functionally associated with muscle tissues in the reproductive and digestive systems [46,47]. In fact, in the flatworm *Mesostoma ehrenbergii* (Rhabdocoela: Platyhelminthes), the exogenous administration of AT (10^-14^ and 10^-12^ M) induced muscular contractions, particularly in the pharynx, suggesting that it acts as a myoregulator [47]. 

The AT receptor was originally characterized in the silk moth *Bombyx mori* (Lepidoptera: Insecta) as a seven transmembrane domain protein pertaining to the family of the vertebrate orexin receptors [48]. Recently, the receptor was characterized in three additional holometabolous insect species from different orders - Diptera (*Aedes aegypti*) [49]; Coleoptera (*Tribolium castaneum*) [50] and Lepidoptera (*M. sexta*) [51]. Interestingly, the detection of AT in several groups of Protostomata but apparently not in Deuterostomata suggests that this peptide could be a synapomorphic feature of Protostomata. Indeed, the presence of AT in organisms that do not undergo metamorphosis suggests that it was originally involved in myotropic activities, the induction of the synthesis of JHs being a secondary function [47]. As a first approach to understand the evolutionary origin of this pleiotropic peptide, we decided to investigate the expression of AT-like peptides in *Hydra plagiodesmica* (Hydroazoa: Cnidaria). A combination of immunohistochemistry using AT-antibodies and AT-Qdot nanocrystal conjugates was used to identify immunoreactive nerve cells containing the peptide and epithelial-muscular cells targeted by AT. Physiological assays using AT and AT- antibodies revealed that while AT stimulated the extrusion of the hypostome in a dose-response fashion in starved hydroids, the activity of hypostome in hydroids challenged with food was blocked by treatments with different doses of AT-antibodies. Immunochemical, physiological and bioinformatics evidence suggest that AT is an ancestral neuropeptide involved in myoregulatory activities associated with meal ingestion and digestion. 

## Materials and Methods

### Animals

*Hydra plagiodesmica* hydroids were obtained from a colony maintained in dechlorinated water at 20±2°C with a 12:12 hour light/dark period. Animals were fed *Artemia salina* until they were processed. For physiological experiments, specimens were starved during the 72 hours prior to the beginning of the experiment. All the experiments included groups of 6 individuals for each treatment. Each specimen was kept isolated throughout the experiment. 

### Immunohistochemistry and phalloidin labeling

Hydroids were fixed in formaldehyde-phosphate buffered saline (PBS) (4%) at 4 °C for 12 h. They were then washed in PBS-Tween (0.05%) (PBS-T), permeabilized in Triton X-100 (1%) (24 h at 4 °C) and blocked with 3% bovine serum albumin (BSA) for 2 h. Materials were then incubated overnight at 4°C with a polyclonal antiserum developed against allatotropin of the mosquito *Aedes aegypti* (1/500 in 3% BSA diluted in PBS-T). The antibody recognizes *A. aegypti* AT with the following sequence of amino-acids: APFRNSEMMTARGF [28]. The specificity of antibody binding was previously demonstrated by preadsorption with the cognate peptide, both in insect species [36,37,52] as well as in other invertebrate groups [46,47]. Hydroids were then incubated with a FITC-labeled goat anti-rabbit secondary antibody (Santa Cruz Biotechnology) (1/1000 in blocking-buffer) for 24 h at 4°C. To visualize the arrangement of the epithelial-muscular cell fibers, samples were co-incubated with a rhodamine-phalloidin solution (Sigma–Aldrich) (1/1000) [46,47]. After every step, hydroids were washed (3 times x 20 min) with PBS-T (0.05%). As controls, the incubation with the primary antibody was replaced with PBS. Specimens were mounted with Vectashield mounting medium (Vector Laboratories, Burlingame, CA) and analyzed with a Laser Scan Confocal Microscope Zeiss LSM 510 Meta.

### Quantum dot-biotinylated peptide conjugates

Quantum dot nanocrystals (Qdot) conjugated to streptavidin (1 µM) were purchased from Quantum Dot Corporation (Hayward, CA). Their peak of emission is at 605 nm (red). Biotinylated *A. aegypti* AT and Allatostatin-C (AST-C) were custom synthesized (Biopeptide, San Diego, CA). Each Qdot crystal is conjugated to 5-10 streptavidin molecules, with a total of 20-40 binding sites for biotin. Each biotinylated neuropeptide stock was dissolved at 0.92 mM in 100% DMSO (Dimethylsulfoxide, Sigma) and stored at -70 °C. An excess of neuropeptides (4.3 µl) was added to 10 µl of QDs, along with 58.7 µl of incubation buffer (2% BSA in 50 mM borate buffer pH 8.3 and 0.05% NaN3). The mixture was incubated for 1.5 h at RT. To remove the excess of free peptide, Qdot-peptide conjugates were washed five times with 0.5 ml of PBS in a Microcon-100 concentrator (Amicon Bioseparations, Bedford, MA, USA) at 10,000 rpm 5 min. The Qdot-peptide conjugates were recovered in 100 µl of PBS and stored at 4 °C.

### Qdot-AT binding to tissues

 Hydroids were incubated in a solution containing Qdot-AT conjugates for 30 mins, fixed for 12h at 4 °C in formaldehyde-phosphate buffered saline (PBS) and examined under microscopic at 543 nm. A similar sample was incubated, fixed and processed for AT-antibody immunohistochemistry as described above; allowing for the analysis of the coexistence of cell populations being labeled with the AT-antibody and binding Qdot-AT conjugates. As a control for the Qdot labeling, a third group of individuals was incubated in a solution containing non-conjugated Qdot nanocristals. To further test the specificity of Qdot-AT recognition of the population cells, another group of individuals was incubated with Qdot-AST-C conjugates and processed for microscopy as described above.

### Myotropic assays

To analyze the myoregulatory activity of the AT-like peptide in *H. plagiodesmica*, hydroids were treated with different doses of synthetic *A. aegypti* allatotropin. Six sets of six hydroids were starved for 72 hours and were individually placed in fresh water and acclimated for 10 to 15 min. Once the experimental specimens were acclimated, the solution was replaced with water containing AT at concentrations ranged from 10^-16^ to 10^-8^ M. A control group did not receive peptide. The experimental specimens were examined individually under a binocular microscope and the state of the hypostome (hypostome completely extruded or not) assessed at 3, 5, and 15 min. 

In a second series of experiments, we tested the ability of the *A. aegypti* AT-antiserum to block the hypostome extrusion. Similar groups of starved hydroids were incubated with *Artemia salina* in a solution containing anti-AT (1/100; 1/500; 1/1000; 1/5000 and 1/10000). Control groups did not received antiserum. The experimental specimens were examined individually under a binocular microscope, the position of the hypostome was assessed at 3, 5, 15, 30 and 45 min and the number of individuals showing the hypostome completely extruded was recorded. Data are expressed as percentage of the control.

#### Identification of Allatotropin receptor sequences

A protein BLAST search was performed in GenBank using the sequence of *Manduca sexta* AT- receptor. A similar search was performed within the draft of the *Hydra magnipapillata* genome (http://hydrazome.metazome.net/cgi-bin/gbrowse/hydra/). Sequences sharing a minimum of 27% identity with an *e*-value equal to or less than 1e-23 were then blasted against insect species. Only those sequences that presented all the complete transmembrane domains and shared more similitude between them than with the sequence of the *Drosophila melanogaster* fmrf-receptor (selected as outgroup) were included. A total of 22 sequences pertaining to Cnidaria, Mollusca, Arthropoda, and Nematoda were selected (Table 1). These sequences were aligned using the Clustal Wallis algorithm (http://www.ebi.ac.uk/Tools/msa/clustalw2/) and further analyzed by the JalView 2.7 [53]. A phylogram was constructed using a Neighbor Joining method using the Blocks of Amino Acid Substitution Matrix (BLOSUM62) [54]. 

**Table 1:** Allatotropin receptors identified in Protostomata.

**Name**	**Order/Class**	**Phylum**	**Score**	***e*-value**	**% identity**	**Accession**
*A. aegypti*	Diptera/Insecta	Arthropoda	434	4e-146	53	AEN03789.1
*T. castaneum*	Coleoptera/Insecta	Arthropoda	417	2e-141	54	XP_973738.2
*M. sexta*	Lepidoptera/Insecta	Arthropoda	942	0.0	100	ADX66344.1
*D. plexippus*	Lepidoptera/Insecta	Arthropoda	604	0.0	74	ADX66344.1
*B. mory*	Lepidoptera/Insecta	Arthropoda	585	0.0	73	BAG68415.1
*N. vitripennis*	Hymenoptera/Insecta	Arthropoda	412	5e-140	55	XP_001604582.2
*B. terrestris*	Hymenoptera/Insecta	Arthropoda	412	2e-140	58	XP_003402490.1
*B. impatients*	Hymenoptera/Insecta	Arthropoda	414	2e-142	58	XP_003486747.1
*A. florea*	Hymenoptera/Insecta	Arthropoda	414	7e-141	52	XP_003690070.1
*M. rotundata*	Hymenoptera/Insecta	Arthropoda	376	2e-142	60	ADX66344.1
*H. saltator*	Hymenoptera/Insecta	Arthropoda	414	5e-126	54	EFN76143.1
*S. gregaria*	Orthoptera/Insecta	Arthropoda	407	4e-141	56	AEX08666.1
*R. prolixus*	Hemiptera/Insecta	Arthropoda	389	6e-135	63	Pending
*C. elegans*	Rhabditida/Secementea	Nematoda	146	8e-39	33	NP_493283.3
*C. brenneri*	Rhabditida/Secementea	Nematoda	145	2e-38	35	EGT31251.1
*C. briggsae*	Rhabditida/Secementea	Nematoda	132	8e-34	35	XP_002646062.1
*C. teleta*	Polychaeta/Capitellida	Annelida	324	9e-105	47	ELU16138.1
*C. gigas*	Ostreoidea/Bivalvia	Mollusca	279	6e-89	46	EKC31663.1
*H. magnipapillata*	Anthomedusae/Hydrozoa	Cnidaria	127	7e-33	30	XP_002163508.1
*H. magnipapillata*	Anthomedusae/Hydrozoa	Cnidaria	114	3e-28	28	XP_004205906.1
*H. magnipapillata*	Anthomedusae/Hydrozoa	Cnidaria	100	4e-23	27	XP_002159553.1
*T. adherens*	-----------------------------------	Placozoa	124	4e-31	27	XP_002117931.1

The table includes the score, *e*-value, percentage of identity and accession numbers.

### Statistics

Differences between treatments were analyzed by two sample t-test between percents analysis. Only differences equal or less than 0.05 were considered significant.

## Results

### AT-like expression in Hydra plagiodesmica

**Figure 1:**
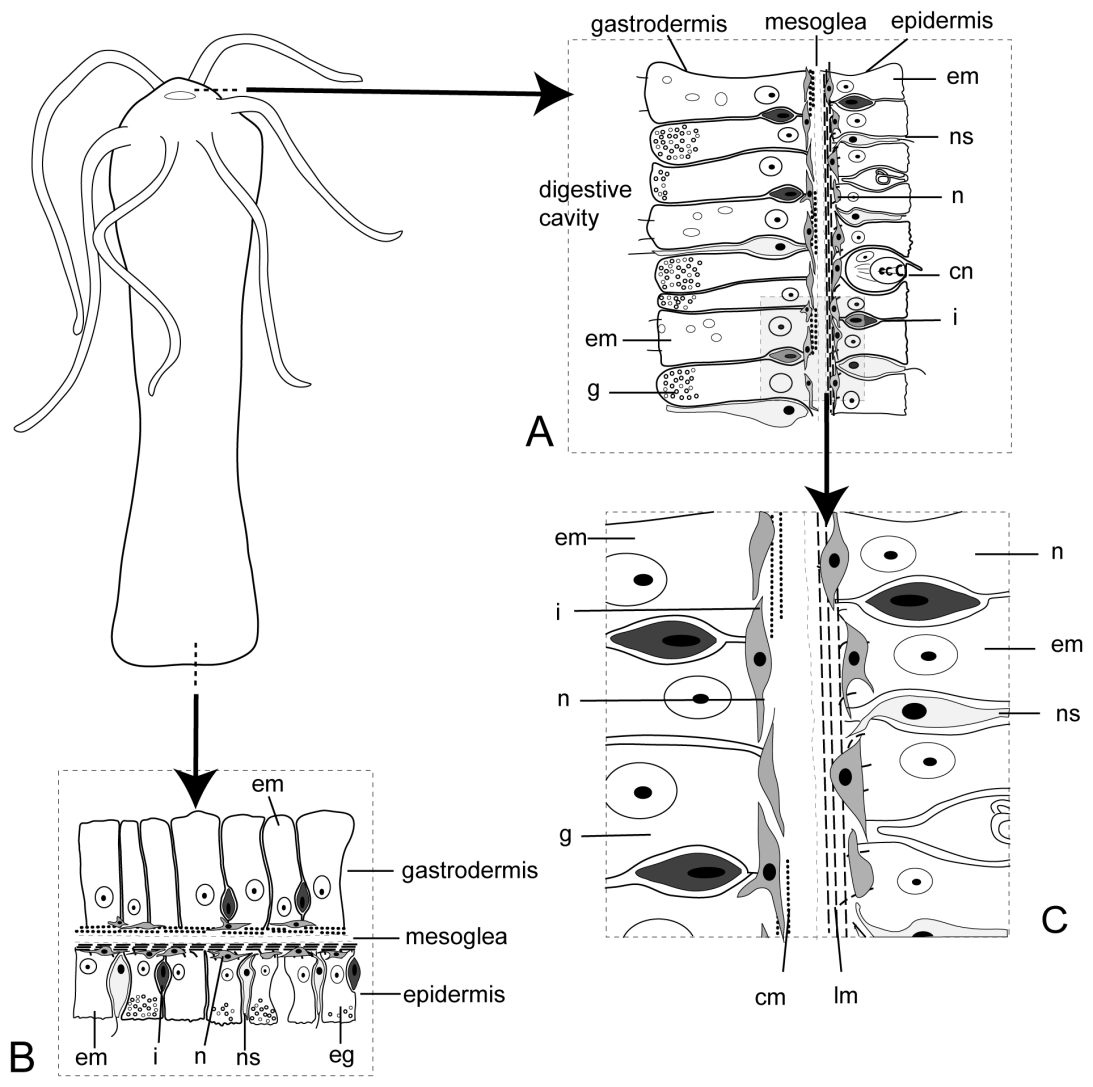
Schematic representation of *Hydra* sp. Cross sections of two regions of the hydroid body. **A**: hypostome. **B**: pedal disk. **C**: detail of the nerve cells forming a net on the base of the epidermis and gastrodermis. **cm**: circular muscular layer formed by the contractile extensions of the nutritive muscle cells; **cn**: cnidocito; eg: epithelial gland cell; **ep**: epithelial-muscular cell; **g**: mucous and enzymatic gland cell; **i**: interstitial cell; lm: longitudinal muscle layer formed by the contractile extensions of the epithelial-muscular cells; **n**: nerve cell and **ns**: neurosensory cell.

**Figure 2:**
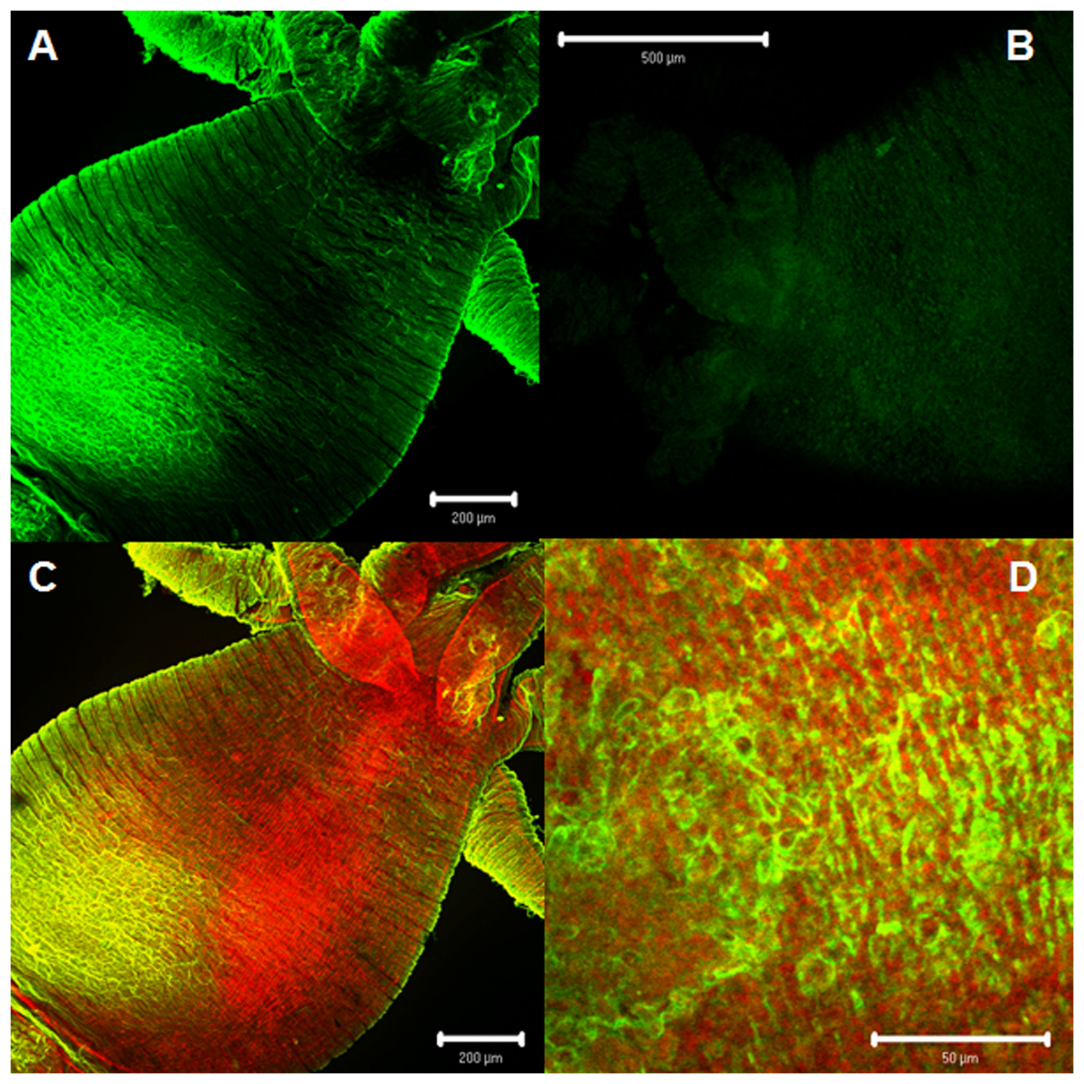
AT-like immunoreactivity. **A**: Panoramic view of a specimen labeled with anti-AT antiserum showing the presence of immunoreactive material in different parts of the body. **B**: Similar view in which the primary antibody incubation was replaced for saline solution. **C**: Similar view as in **A** showing the co-existence of f-actin filaments and immunoreactive material **D**: A magnified view showing the spatial relationship between AT-like nerve cells and epithelial-muscular cells. Motor nerve cells (green) and epithelial-muscular cells labeled with phalloidin (red).

**Figure 3:**
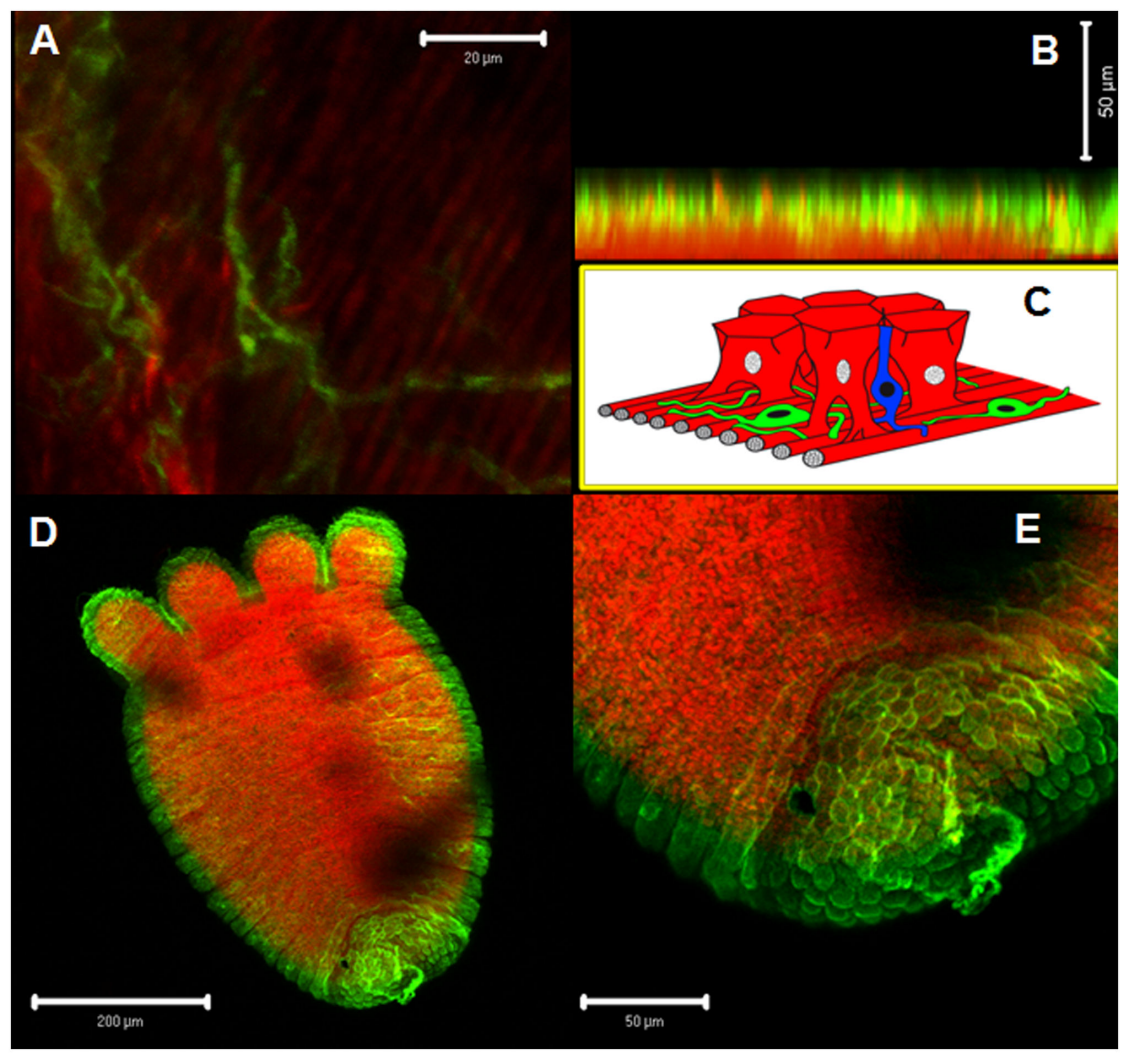
AT-like immunoreactivity. **A**: confocal 3D reconstruction showing AT-like nerve cells in a net-like arrangement and f-actin filaments. **B**: confocal 3D reconstruction of a cross-section of the hydroid body wall showing the anatomical relation between AT-like motor nerve cells and epithelial-muscular cells, as well as the colocalization of the cells producing the peptide, with contractile cells. **C**: Schematic representation of **B** including sensory nerve cells (blue) (modified from [69]). **D** and **E**: Panoramic (**D**) and detailed view (**E**) of an hydroid showing the presence of allatotropic-like cells in the pedal disc. Motor nerve cells (green) and epithelial-muscular cells labeled with phalloidin (red).

Figure 1 shows a generic scheme of a hydroid, with detailed views of the cellular organization at different regions of the body. The immunohistochemical analysis showed the existence of several AT-immunoreactive cell populations distributed throughout the hydroid body. The absence of immunoreactivity in control suggests that the labeling is specifically originated by recognition of an AT-like peptide (Figure 2A and B). The simultaneous use of the AT-antibody and phalloidin labeling demonstrated the presence of AT-like immunoreactive nerve cells that were anatomically related with the epithelial-muscular cells (phalloidin-labeled) present in the stalk (Figure 2C). A magnified view showed that allatotropic-like cells presented cytoplasmic projections running between epithelial-muscular cells that were circularly distributed (Figure 2D). The images in Figure 3A and B represent a 3D confocal reconstruction of the stalk’s cells; showing a detailed view of the morphological relationship between epithelial-muscular and nerve cell populations. Moreover, the close localization of cells producing the peptide (nerve cells) and cells containing f-actin filaments (epithelial-muscular cells) was shown by the co-localization of both signals in the same area (Figure 3B and C). The presence of a similar allatotropic-like cell population was detected in the pedal disc (Figure 3D and E).

### AT-Qdot labeling

**Figure 4:**
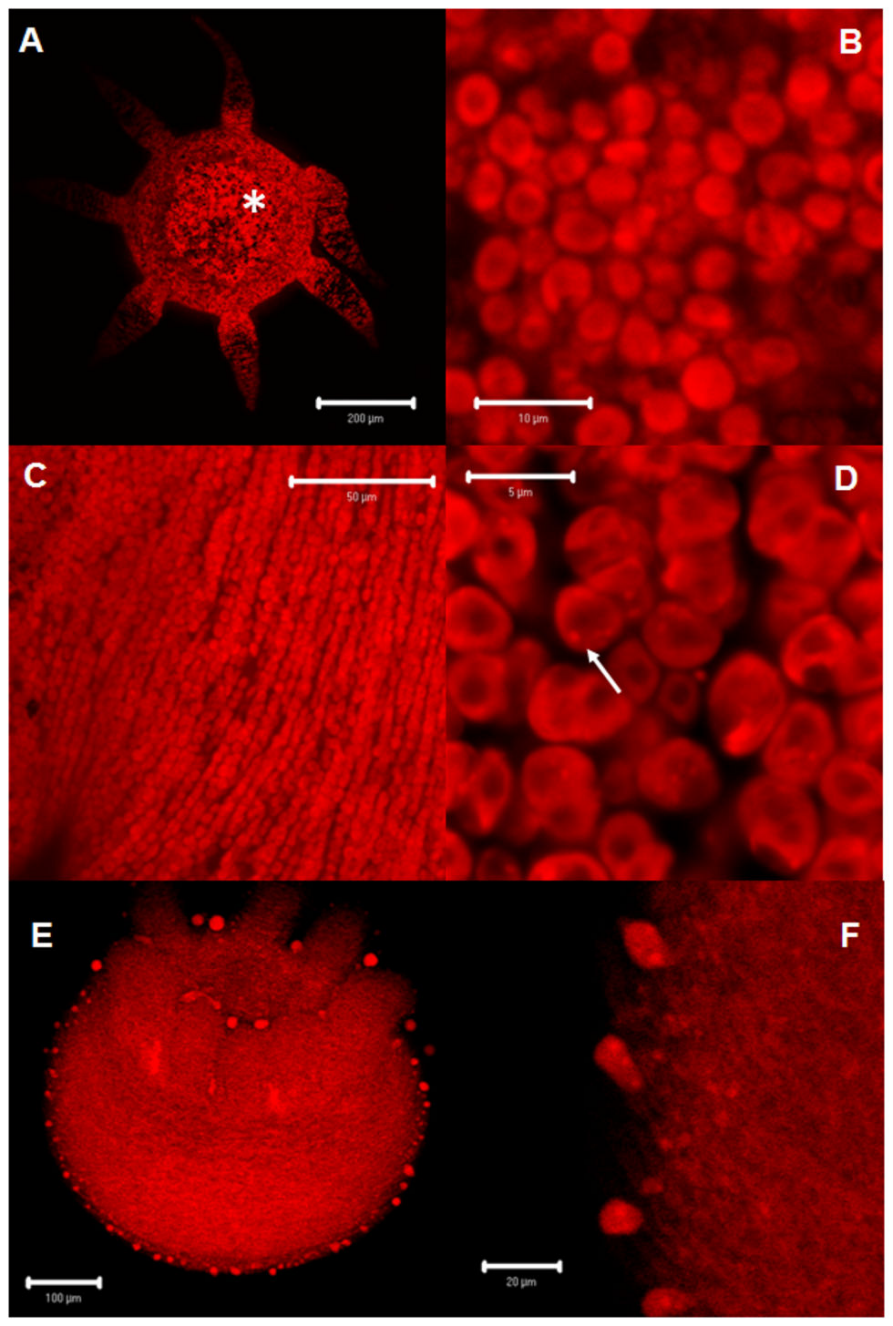
Qdot-AT and AST-C labeling and AT-immunoreactivity. **A**: Pedal disc view showing the distribution of cells labeled with Qdot-AT (_*_) **B**: Magnified view showing the cells in clusters **C**: View of the body wall of a hydroid showing AT recognizing epithelial-muscular cells at the stalk, as well as the distribution of the cells in a circular arrangement **D**: Magnified view of the epithelial-muscular circular cells at the stalk. Note the cells in two circular rows, as well as the presence of endocytic vesicles (arrow). **E**: View of the body wall of a hydroid showing AST-C recognizing epithelial-muscular cells at the stalk. Note that the distribution of the labeled cells is different to the labeling corresponding to the AT peptide. **F**: Magnified view of the same epithelial-muscular circular cells, also showing endocytic vesicles.

**Figure 5:**
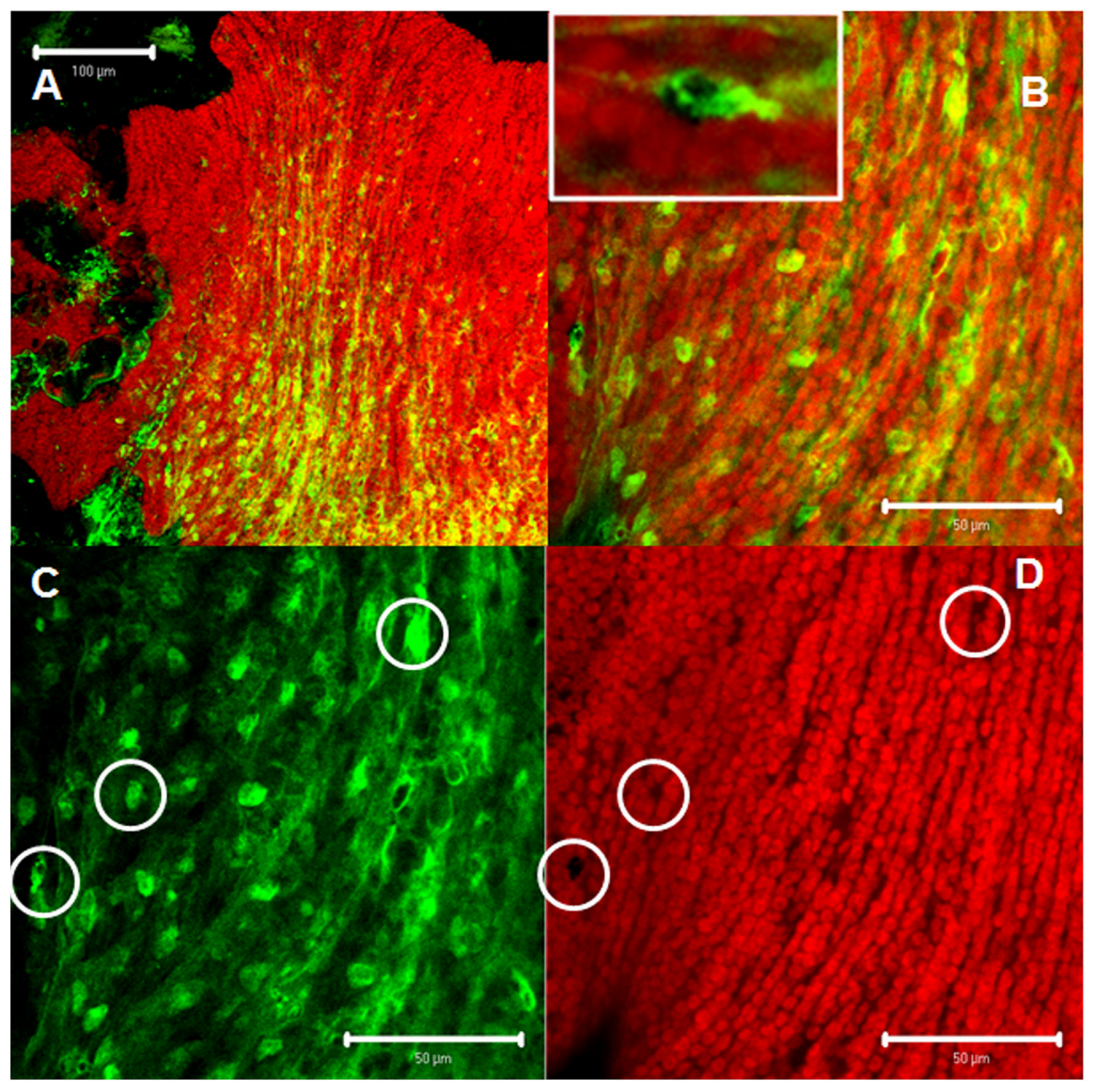
Qdot-AT labeling and AT-immunoreactivity. **A**: Panoramic view at the bottom of the stalk showing the pedal disc of a hydroid. **B**: Magnified view of the stalk suggesting. a morpho-functional relationship between both cell populations. **Inset**: detailed view of a nerve cell. Note that the morphology and spatial orientation of the cell and its projection clearly resembles the motor nature of the nerve cells. **C** and **D**: show both channels independently. Note that the position of nerve cells (**C**), clearly corresponding with non-labeled spaces in (**D**) (open circles), showing that nerve cells are intercalated between epithelial-muscular cells recognizing AT peptide and reinforcing the morpho functional relationship between these two populations. Epithelial-muscular cells recognizing AT-Qdots conjugates (red) and AT-like motornerve cells (green). Colocalization of markers (yellowish green).

The microscopic analysis showed that after 30 min of incubation AT-Qdot conjugates were bound to *H. plagiodesmica* cell populations expressing putative AT receptors. The incubation of hydroids in a solution containing unconjugated Qdot nanocrystals did not generate a signal, suggesting that the binding to cells is caused by the recognition of the AT portion of the conjugated molecules. The distribution of the cell populations that bound the AT-Qdot conjugates were identical to those detected in the pedal disc and stalk using phalloidin, suggesting the mioepithelial nature of these cells (Figures 2C, 3C, D, 4A, 4B and 5A) and also showing a spatial coincidence with AT-immunolabeled cells. Notably, the distribution of immunolabeled populations of cells at the pedal disk and on the stalk were different (Figure 3), forming clusters in the pedal disk (Figure 4A and B) and groups of cells circularly orientated in the stalk (Figure 4C and D). Furthermore, when hydroids were incubated with AST-C-Qdot conjugates, the distribution of the labeled cells was clearly different from those labeled with AT-Qdots, suggesting that Hydra’s cells recognized specifically different peptides (Figure 4E and F). When hydroids were processed to detect both AT immunoreactivity and AT-Qdots binding, the microscopic analysis showed a clear concordance between the stalk cells producing the peptide and the population of cells recognizing the AT-Qdot conjugates (Figure 5A and B); indeed, in a magnified view, the motor nerve cells type morphology of the AT-like cells is evident (inset in Figure 5B). AT-Qdot conjugates were localized inside cytoplasmic vesicles, suggesting that the complex was internalized probably by means of AT receptor recognition (Figure 4D). When both channels (immunohistochemistry label in green and Qdot label in red) were analyzed separately we could observe that motor nerve cells and their projections were intercalated running along spaces among epithelial-muscular cells (Figure 5C and D).

### Allatotropin-like peptide myotropic activities regulating feeding

**Figure 6:**
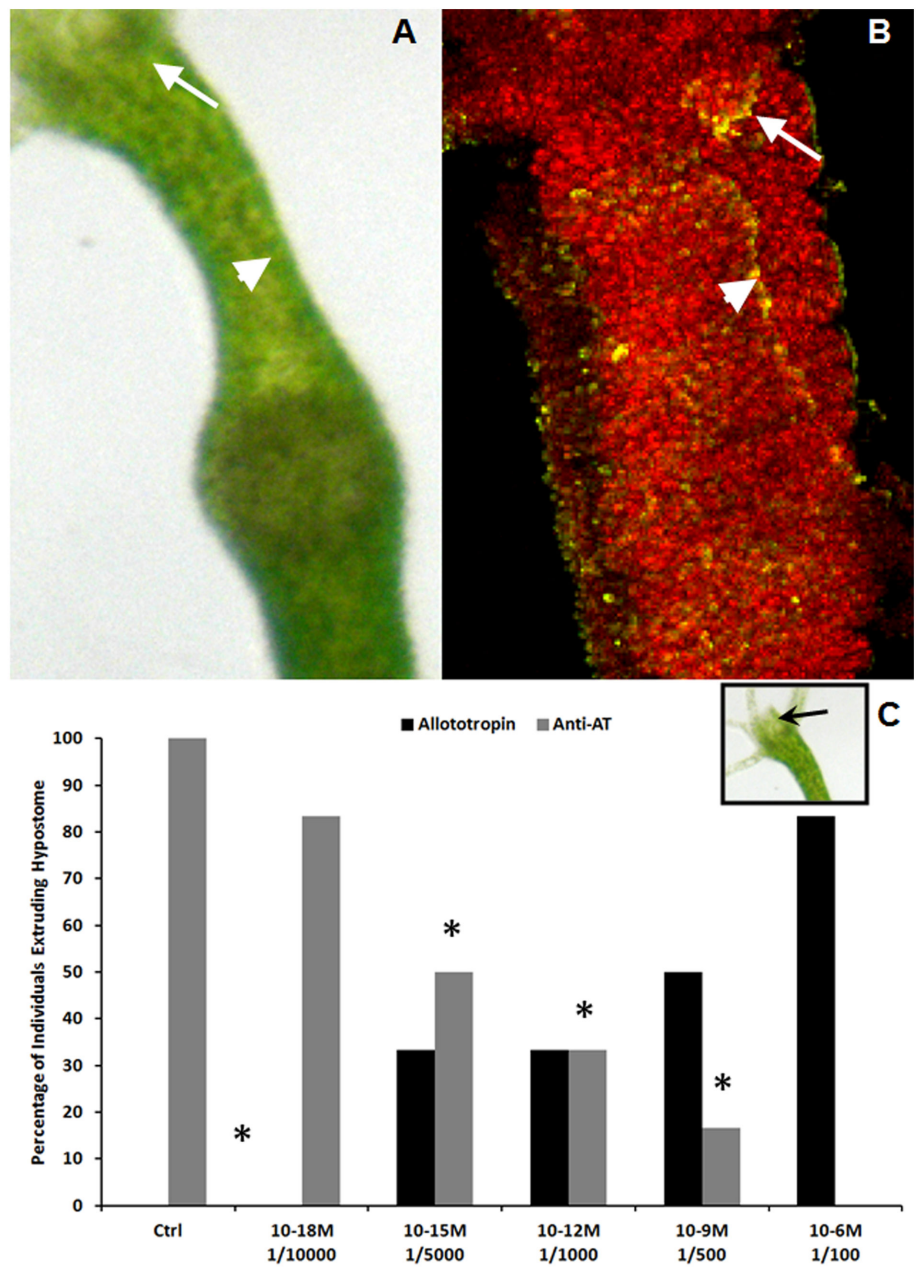
Myotropic activity of AT-like peptide and feeding. **A**: Specimen of *H. plagiodesmica* after the ingestion of an *A. salina* egg. **B**: confocal 3D reconstruction of an hydroid after double labeling with AT-antiserum (green) and nanocristals (red). Note the colocalization of both markers at the level of the hypostome (arrows) and gastroenteron (arrow heads). **C**: Physiological assay demonstrating the AT involvement in feeding movements. Black columns: Hypostome response of 72 h starved hydroids to treatment with different doses of AT. Grey columns: Hypostome response of 72 h starved hydroids challenged with food and exposed to different doses of AT-antiserum. The results are expressed as percentage of individuals that completely extruded the hypostome. (*): Significant differences between hydroids treated with AT or AT-antiserum and controls.

Hydroids responded to the presence of food by extruding the hypostome to capture and ingest the prey. To analyze the response of the hypostome epithelial-muscular cells to AT, we studied the dose-dependent effect of AT on groups of 72 hours starved hydroids. Our results showed that the hypostome reacts to AT in a dose-response manner (Figure 6C). 

The role of AT as stimulant of hypostome activity was confirmed by an independent set of experiments where AT-antiserum was utilized to block the response of the hypostome to the presence of food. The control group, which did not receive treatment with the AT-antiserum, showed the normal extruding behavior of the hypostome; while groups treated with different concentrations of the AT-antiserum showed a dose-response inhibition of hypostome activity, suggesting that the movement of the hypostome was specifically blocked by the antiserum (Figure 6C).

### In silico search for allatotropin receptors in invertebrates

**Figure 7:**
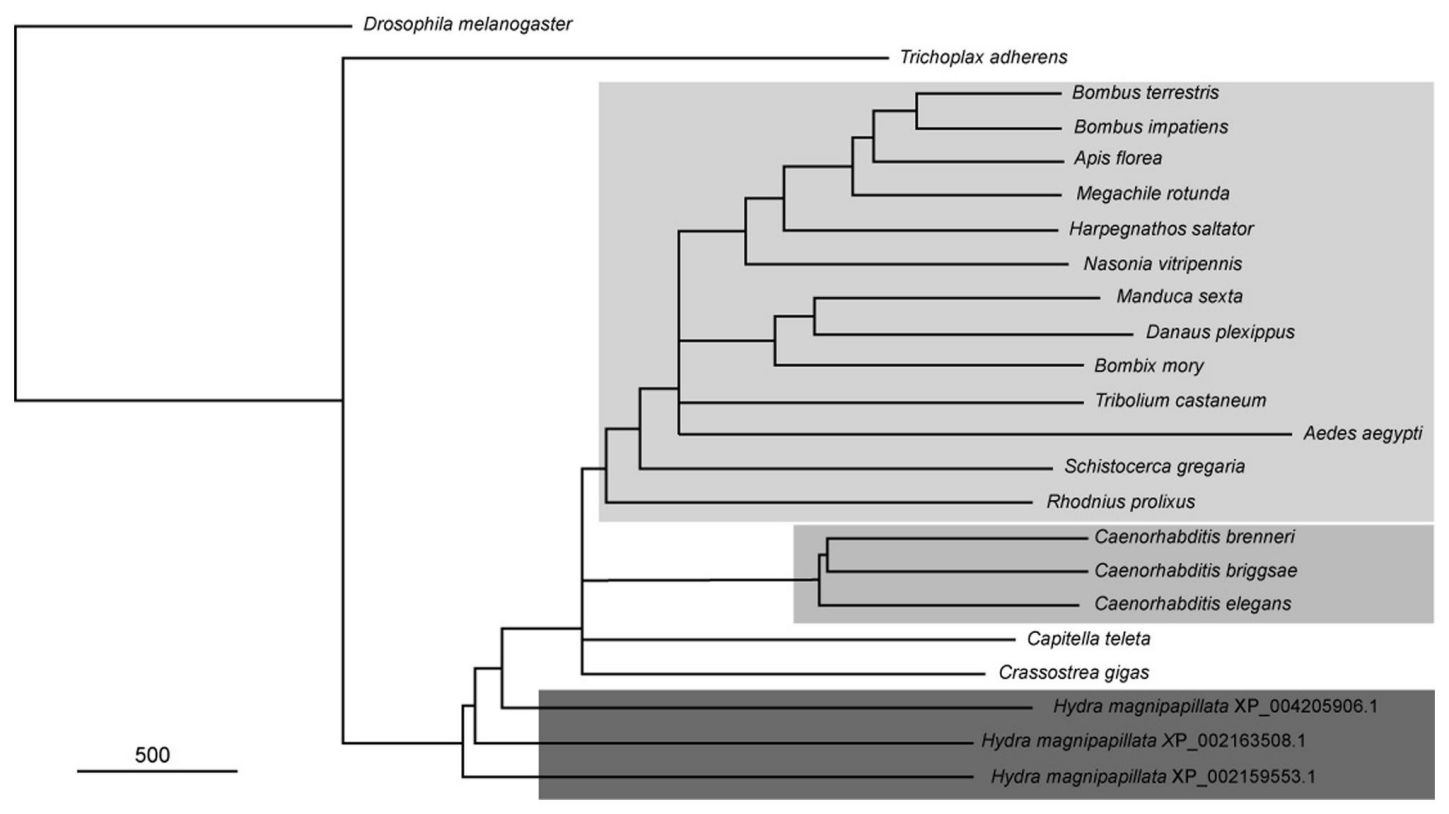
Phylogram of AT-receptor. Species from the same phylum are labeled by a similar gray tone blocks. The tree is enrooted using the *D. melanogaster* FMFRamide receptor [50].

We identified a total of 22 sequences corresponding to Arthropoda, Cnidaria, Nematoda, Placozoa and Mollusca that shared significant similarity with the *M. sexta* AT receptor (Table 1). The alignment of all these sequences is shown in Figure S1. A phylogenetic analysis showed that all the holometabola insect sequences (orders Hymenoptera, Lepidoptera, Coleoptera and Diptera) cluster together on a branch in close proximity with the only two hemimetabolous species identified (orders Hemiptera and Orthoptera). The closer group to the Arthropoda was composed by three species of the genus *Caenorhabditis* sp. (Nematoda). The Ecdysozoa group (Arthropoda and Nematoda) were close to the Mollusca and Annelida species, conforming all together the bilateria phyla represented in the tree. Non-bilateria phyla (i.e. Cnidaria and Placozoa) appeared in the base of the other groups. Regarding cnidarians, they included three sequences of *H. magnipapillata* sharing 30, 28 and 27 % of identity (access numbers: XP_002163508.1; XP_004205906.1; XP_002159553.1). Beyond the identity, further analysis of the sequences showed similarity indices of 50, 50 and 49 % respectively, suggesting a high conservation of the whole protein, as well as of the transmembrane domains, where the indices were higher (data not shown). Finally, *T. adhaerens* that showed 27% of identity and 47% similarity, shares a common ancestor with the rest of the metazoan groups included in the analysis (Figure 7). 

## Discussion

Our results provided evidence for the existence of an AT-like peptide in hydroids of *H. plagiodesmica* (Cnidaria: Hydrozoa). Labeling with AT-specific antibodies revealed immunoreactivity in cells located in the stalk that clearly resembled nerve cells. The spatial distribution of these allatotropic-like cells, intercalated between epithelial-muscular cells, suggested the existence of anatomical and functional relationships between these two cell populations and advocates for myoregulatory functions of AT-like molecules in *Hydra* sp.

Qdot technology has been previously used in *Hydra vulgaris* to demonstrate GSH-mediated, highly specific, binding of Qdot-GSH conjugates to the gastric region; which indicated the presence of GSH binding proteins in the endodermal cell layer [55]. Our results showed that the cells that bound the AT-Qdots conjugates were localized in the same stalk and pedal disc regions in which AT-like cells were present; suggesting again the existence of a functional relationship between the nerve cells producing the peptide and the targeted epithelial-muscular cells. The two different nanocrystal conjugates (AT and AST-C) were internalized by different target cells and detected inside endocytic vesicles, strongly suggesting the existence of receptors that specifically recognized the peptides in *H. plagiodesmica*. 

The combined treatment of hydroids with AT-Qdots and AT-antibody revealed the coincidence of both signals in the mouth region of the hypostome, as well as in the wall of the coelenteron. The distribution of AT-immunoreactive cells, along with the dose-response stimulation of feeding by AT, suggests that the AT-like peptide could regulate myotropic activities critical for the ingestion and digestion of meals. The cnidarians are the simplest organisms in which movements involved in feeding behavior are governed by a neuromuscular system [56]. The body column of the hydra during feeding undergoes a complex series of movements resembling those performed by organisms with a fully developed digestive system [57]. Comparison of the movements to those in mammals showed similarities in appearance to esophageal reflex, segmentation movement, and defecation reflex. When nerve cells were eliminated, polyps showed only a weak segmentation movement, demonstrating that the diffuse nerve net in the body column of *Hydra* sp. primarily regulates the movements just as the netlike enteric nervous system does in mammals [57]. Acetylcholine and serotonin, which are regulating similar movements in the mammalian enteric system, were not involved in *Hydra* sp. [55]. Starved hydroids of *H. plagiodesmica* showed similar movements when they were exposed to *A. salina* or stimulated by the treatment with the synthetic AT. These feeding movements were inhibited in a dose-dependent fashion when individuals were treated with anti-AT antiserum. In fact, regurgitation, a movement which normally occurs between 6-9 hours after food ingestion [57], was evident in several hydroids around 30-40 min after treatment with AT, suggesting again that the AT-like peptide is involved in the process of ingestion. 

The physiological responses induced by AT, as well as the specific interactions of cell populations with Qdot-AT conjugates, suggested the presence of an AT-like receptor in *H. plagiodesmica*. Our search for orthologues of AT-receptors in invertebrates detected 22 sequences from several phyla including Placozoa, which is probably the most ancient group of Metazoa. The distance tree representing the relationships between sequences of the AT receptor family was coherent with recognized phylogenetic relationships between species. Receptors from insect species (Arthropoda) were closely related with those from Nematoda; these two groups together with another six phyla share a common ancestor, forming the monophyletic group of the Ecdysozoa [58,59]. Receptors from Mollusca and Annelida, two groups in the Lophotrochozoa, are related. Finally, the sequences pertaining to Cnidaria and Placozoa are well resolved as independent groups appearing as ancestors of the bilateria phyla. The evolutionary position of the Placozoa phylum is still controversial but modern molecular and genetic studies support its position as a basal lower metazoan phylum [60,61]. This is consistent with the position of the AT-receptor in this tree, and suggests that AT-receptors already appeared in the more primitive group of Metazoa. 

Peptidic messengers play fundamental roles acting both as local neuromodulators, neurohormones and also in an endocrine and paracrine mode; being secreted not only by neurons, but also by epithelial cells. They likely comprise the most ancient group of neurotransmitters [7]. In this context, the Allatotropin/Orexin family of receptors would constitute a group of proteins broadly distributed from Placozoa to Chordata. The peptides associated with these receptors (allatotropins and orexins) are not chemically or functionally related. Orexins (also called hypocretins) are 28 to 33-amino-acid peptides mainly involved in the control of feeding, sleep-wakefulness, neuroendocrine homeostasis and autonomic regulation [62,63]. On the other hand, allatotropins constitute a family of peptides originally characterized based on its ability to activate JH synthesis [21], but later demonstrated to be pleiotropic, inhibiting ion transport [29], inducing the secretion of gut enzymes [30], as well as been involved in circadian rhythms control [40-43]. Due to the difficulties of analyzing highly diverse and repetitive pro-peptides, the relationships among different families of peptides could be elusive. In fact, an orthologous relationship between allatostatin-A and galanin which is not evident at this level is supported by the orthology of their receptors and similar roles [64]. Our search of Allatotropin peptide or allatotropin precursor in *H. magnipapillata* genome did not show the presence of orthologous peptides, but demonstrated the presence of sequences corresponding to G-protein coupled receptors sharing 30% of identity and 50% of similarity with *M. sexta*. Furthermore, a search for orexin homologous did not reveal any significant match in Protostomata and the same was true when we searched for allatotropin related peptides in Deuterostomata; suggesting that even if both families of peptides share related receptors, the ligands evolved independently in these two groups. In fact the possibility that allatotropin/orexin and their receptors are orthologous representing an ancient bilaterian family was recently suggested [64].

The original function of AT as a myoactive regulator has been already previously proposed [65]. The presence of genes coding for AT-like peptides in Annelida and Mollusca [44,45], as well as the existence of cells producing AT-like peptides in Platyhelminthes has also been recently proposed [46,47]. Cnidarians are considered basal to the bilateral animals, being the first group which exhibits a neuronal tissue, which could be seen as the starting point in the evolution of the central nervous system [66]. Our results suggest that an AT-like peptide is involved in the feeding process in *H. plagiodesmica*; and its activity could be mediated by receptors members of the Allatotropin/orexin family. The myoregulatory activity of AT on the gut has been described in several insect species [23,31,32,34-39], as well as its cardioacceleratory effects [33,34,38,39]; suggesting that the ancient myoregulatory function has been maintained along the evolutionary process. Interestingly, it was recently shown that orexins also could have myoregulatory functions on the smooth muscle of the mouse duodenum [67], supporting the idea that this was the primitive function of these peptides and their receptors [7].

G-protein coupled receptors are members of the largest, ubiquitous and most versatile family of cell membrane receptors. These types of receptors are also present in unicellular eukaryotes such as yeast [68]. The presence of AT-like receptors in Placozoa, probably the most ancient group of Metazoa, suggests that AT-receptors are a group of ancestral seven-transmembrane receptor originally involved in activities associated with feeding and digestion. As these activities are common and basic processes in any multicellular organism, we would like to suggest that AT and its receptors are in the base of the evolutionary origin of the message-receiver molecular systems based on G-protein receptors.

## Supporting Information

Figure S1 **Alignment of 22 sequences corresponding to Arthropoda, Cnidaria, Nematoda, Placozoa and Mollusca that shared significant similarity with the *M. sexta* AT receptor**.
(TIF)
