# Supplementary figures and images for: Allatotropin: An Ancestral Myotropic Neuropeptide Involved in Feeding
Source: PLoS One. 2013 Oct 15;8(10):e77520. doi: 10.1371/journal.pone.0077520 (PMC3797082; doi:10.1371/journal.pone.0077520)

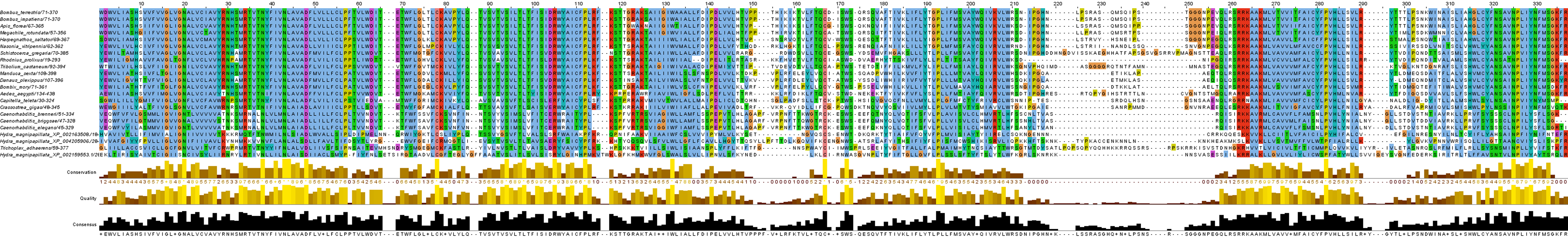

Supplement: Figure S1 — Alignment of 22 sequences corresponding to Arthropoda, Cnidaria, Nematoda, Placozoa and Mollusca that shared significant similarity with the M. sexta AT receptor. (TIF) [file pone.0077520.s001.tif]
